# *Cyclorhizapuana* (Apiaceae), a new species from Sichuan, China

**DOI:** 10.3897/phytokeys.182.67009

**Published:** 2021-09-20

**Authors:** Jing Zhou, Jun-Mei Niu, Xin-Yue Wang, Pei Wang, Ming-Jia Guo, Zhen-Wen Liu

**Affiliations:** 1 School of Pharmaceutical Science and Yunnan Key Laboratory of Pharmacology for Natural Products, Kunming Medical University, Kunming 650500, China Kunming Medical University Kunming China; 2 Yunnan Academy of Forestry and Grassland, 650201, Kunming, Yunnan, China Yunnan Academy of Forestry and Grassland Kunming China

**Keywords:** Apiaceae, *
Cyclorhiza
*, new species, nrITS, phylogeny

## Abstract

A new species, *Cyclorhizapuana* J. Zhou & Z.W. Liu (Apiaceae) from Sichuan Province of China, is described and illustrated here. Morphological comparisons with congeneric species revealed that it is distinguished by its slender habit, sparse annular scars, 4-pinnatisect leaf blade with ultimate segments linear (2–4×0.5–1 mm), subequal rays, oblong fruits with slightly thickened ribs, obconic stylopodium and slightly concave seed face. A molecular analysis based on nuclear ribosomal DNA internal transcribed spacer (ITS) sequences indicated that *C.puana* is genetically distinct from the other two species of the genus. A distribution map, as well as an updated key, are provided for the species of *Cyclorhiza*.

## Introduction

*Cyclorhiza* M.L. Sheh & R.H. Shan is a small genus of Apiaceae subfamily Apioideae, with two species currently recognised (Sheh and Shan 1980; Sheh and Watson 2005; Pimenov 2017). It is distributed in southwest China and characterised by carrot-like roots with prominent annular scars, bracts and bracteoles usually absent, yellow petals and fruits subpentagonal in cross section (Sheh and Shan 1980; Sheh and Watson 2005). Previous phylogenetic studies indicated that the two species of *Cyclorhiza* constituted a highly-supported monophyletic clade in the tribe Komarovieae and showed a sister group relationship to *Calyptrosciadium* Rech. f. & Kuber from SW Asia (Zhou et al. 2009; Downie et al. 2010; Zhou et al. 2020).

During a botanical survey to examine Apiaceae in Sichuan Province of China, we discovered a small population of *Cyclorhiza*, whose morphology was clearly distinct from the other species of the genus. We checked all the collections of *Cyclorhiza* at PE and KUN, and digital resources from CVH and GBIF. We noticed a specimen identified as *C.waltonii* in CSH, whose morphology is exactly the same as in our collection. Further examination of morphological characters, coupled with molecular evidence, convinced us that this plant is a distinct new species, which is described and illustrated here.

## Materials and methods

### Morphological studies

**Figure 1.**
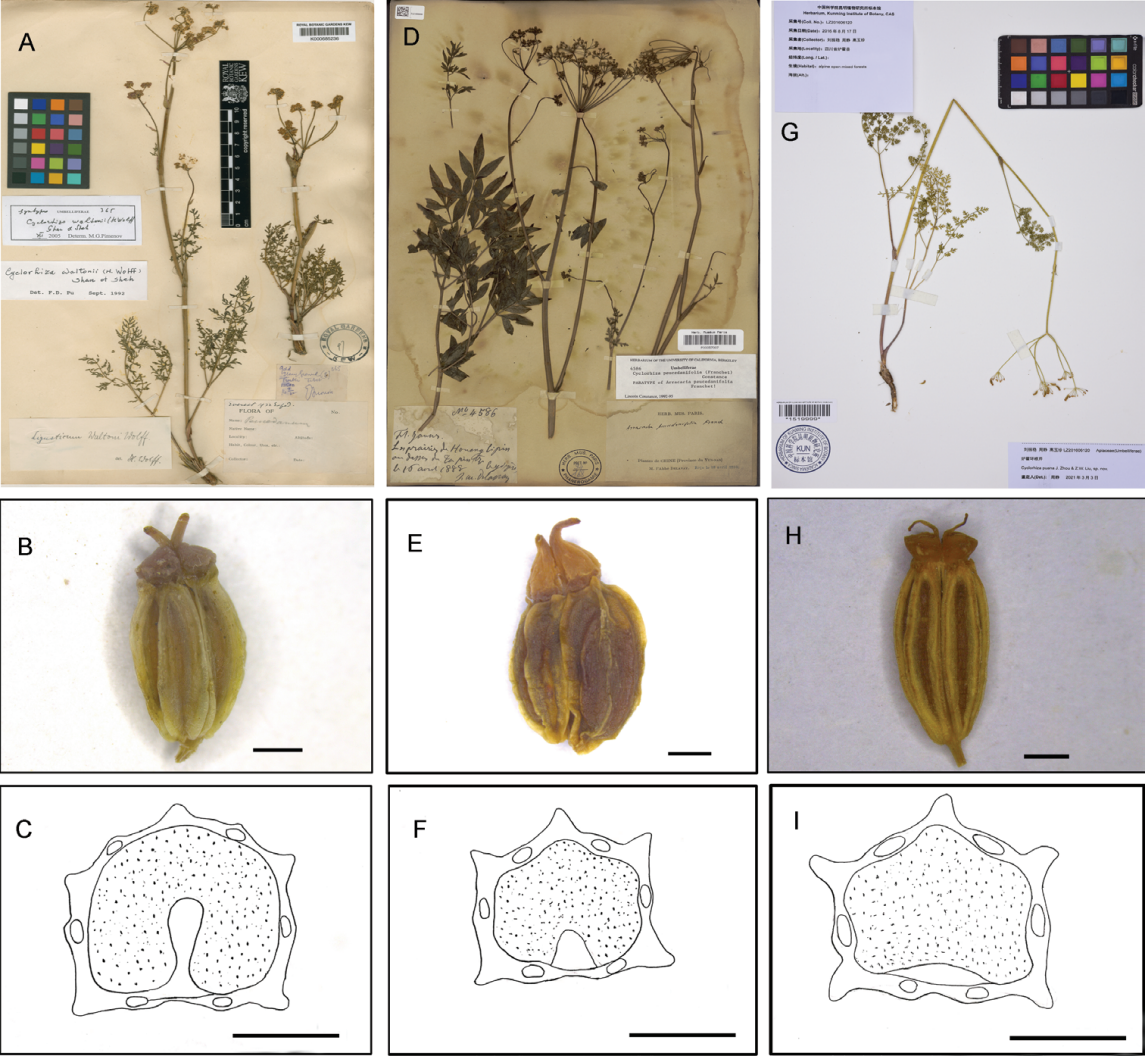
*Cyclorhizawaltonii* (H.Wolff) M.L. Sheh & R.H. Shan (**A–C**) **A** syntype (K000685236) **B** fruit image **C** cross-section; *C.peucedanifolia* (Franch.) Constance (**D–F**) **D** syntype (P00057007) **E** fruit image **F** cross-section; *C.puana* J. Zhou & Z.W. Liu (**G–I**) **G** holotype (KUN1519999) **H** fruit images **I** cross-section. Scale bar: 1 mm.

The morphological characters were examined based on collected specimens. Fruits were taken from dry specimens and studied using a stereo microscope. Herbarium specimens were deposited at KUN. Morphological comparisons with the related *C.waltonii* (H. Wolff) M.L. Sheh & R.H. Shan and *C.peucedanifolia* (Franch.) Constance are provided in Table 1 and Fig. 1.

**Table 1.** Morphological comparison between *Cyclorhizapuana* and congeneric species.

Character	* C. puana *	* C. waltonii *	* C. peucedanifolia *
Root	Annular scars, **sparse**	Annular scars, dense	Annular scars, dense
Rootstock	**Slender**, up to 5 mm in diameter	Stout, up to 20 mm in diameter	Stout, up to 20 mm in diameter
Stem	**Up to 60 cm tall**, simple or several, unbranched or upper 1–3-branched, 2–3 mm in diameter	Up to 100 cm tall, simple, branched above, 2–7 mm in diameter	Up to 150 cm tall, simple, branched above, 10–15 mm in diameter
Leaf	Triangular-ovate in outline, **4-pinnatisect**, **ultimate segments linear**, 2–4×0.5–1 mm	Triangular-ovate in outline, 4-pinnatisect, ultimate segments linear, 4–20 × 2–6 mm	Broadly ovate-triangular in outline, 4-pinnatisect, ultimate segments ovate-oblong to linear-lanceolate, 20–60 × 3–10 mm
Bract	Absent, rarely 1	Absent	Absent, or 1–2
Ray	**4**–**6, subequal**	4–14, unequal	5–12, unequal
Bracteoles	Absent, or 1–2	Absent	Absent
Calyx	Triangular	Triangular	Triangular
Stylopodium	**Obconic**	Low-conic	Low-conic
Fruit	**Oblong**, 5×2 mm	Ellipsoid, 4×2.5 mm	Ovoid, 4–7×2–3.5 mm
Ribs	Filiform, prominent, **slightly thickened**	Filiform, prominent, narrowly winged	Filiform, prominent, narrowly winged
Mericarp	Subpentagonal, seed face **slightly concave**	Subpentagonal, seed face deeply sulcate	Subpentagonal, seed face deeply concave

### Phylogenetic analysis

For the new species, we used our own collection and the specimen deposited as CSH. *Cyclorhizawaltonii* and *C.peucedanifolia* each included two new accessions to examine the possible infraspecific molecular variation (Table 2). In addition, thirty-two ITS sequences were obtained from GenBank to determine the phylogentic position of the new species. The taxa chosen represented a broad cross-section of sampling from the tribe Komarovieae and other clades or tribes of Apioideae, identified in previous phylogenetic studies (Downie et al. 2010; Zhou et al. 2020). Two species of Bupleureae were used to root the tree. The final data matrix comprised a total of 38 accessions. Detailed information about herbarium vouchers, GenBank accession numbers, and literature citations of previously published sequences for those taxa considered in this study are listed in Table 2.

**Table 2.** Detailed information about voucher or source information and GenBank accession numbers for 38 accessions used in phylogenetic analysis.

Taxa	Source/Voucher	GenBank number
*Bupleurumangulosum* L.	Naves and Watson (2004)	AF469008
*B.fruticosum* L.	Naves and Watson (2004)	AF479298
*Calyptrosciadiumpolycladum* Rech. f. & Kuber	Valiejo-Roman et al. (2006)	AY941266 & AY941294
*Chamaesiumparadoxum* H. Wolff	Zhou et al. (2008)	EU236161
*C.thalictrifolium* H. Wolff	Zhou et al. (2008)	EU236162
*C.wolffianum* Fedde ex H. Wolff	Zhou et al. (2008)	EU236163
*Changiumsmyrnioides* Fedde ex H. Wolff	Hu et al. (unpubl.)	DQ517340
*Chuanminshenviolaceum* M.L. Shen & R.H. Shan	Zhou et al. (2009)	FJ385040
*Cyclorhizapeucedanifolia* (Franch.) Constance	China, Yunnan, Niujiaoshan, 261 (KUN)	MW807296
	Zhou et al. (2009)	FJ385042
	China, Yunnan, Lijiang, 1218 (KUN)	MW807297
*C.puana* Zhou & Liu	China, Sichuan, Luhuo, Renda Town, LZ201606120 (KUN)	MW807294
	China, Sichuan, Batang, CSH06561 (CSH)	MW807295
*C.waltonii* (H. Wolff) Sheh & Shan	China, Sichuan, Derong, 31029 (KUN)	MW807298
	China, Sichuan, Derong, 3167 (KUN)	MW807299
	Zhou et al. (2008)	EU236165
*Hanseniamongholica* Turcz.	Katz-Downie et al. (1999)	AF008643 & AF009122
*H.weberbaueriana* (Fedde ex H. Wolff) Pimenov & Kljuykov	Xin & Chen (unpubl.)	JQ936558
*Haplosphaeraphaea* Hand.-Mazz.	Zhou et al. (2008)	EU236167
*Heptapteraanisoptera* Tutin	Valiejo-Roman (unpubl.)	AY941273 & AY941301
*Hymenidiumamabile* (Craib & Smith) Pimenov & Kljuykov	Valiejo-Roman et al. (2012)	FJ469934 & FJ483473
*Hymenidiummieheanum* Pimenov & Kljuykov	Valiejo-Roman et al. (2012)	FJ469951 & FJ483490
*Komaroviaanisosperma* Korovin	Valiejo-Roman et al. (1998)	AF077897
*Parasilausafghanicus* (Korovin) Pimenov	Katz-Downie et al. (1999)	MK088003
*P.asiaticus* Pimenov	Katz-Downie et al. (1999)	AF008642 & AF009121
*Physospermopsisdelavayi* (Franch.) H. Wolff	Zhou et al. (2009)	FJ385056
*Pleurospermopsisbicolor* (Franch.) J. Zhou & J. Wei	Zhou et al. (2014)	KF806587
*P.sikkimensis* C.B. Clarke	Spalik et al. (2010)	GQ379347
*Pleurospermumaustriacum* Hoffm.	Valiejo-Roman et al. (2012)	FJ469962 & FJ483502
*P.cristatum* H. de Boissieu	Li et al. (2011)	JF977828
*Pseudotrachydiumdichotomum* (Korovin) Pimenov & Kljuykov	Spalik et al. (2010)	GQ379342
*P.vesiculosoalatum* (Rech. f.) Pimenov & Kljuykov	Valiejo-Roman et al. (2012)	FJ469964 & FJ483503
*Pterocyclusangelicoides* (Wall. ex DC.) Klotzsch	Valiejo-Roman et al. (2012)	FJ469967 & FJ483505
*P.forrestii* (Diels) Pimenov & Kljuykov	Valiejo-Roman et al. (2012)	FJ469965 & FJ483504
*P.rotundatus* (DC.) Pimenov & Kljuykov	China, Xizang, G18092501 –1 (SZ)	MK078059
*Sphaerosciadiumdenaense* Pimenov & Kljuykov	Terentieva et al. (unpubl.)	FJ489358 & FJ489389
*Tongoloasilaifolia* (H.de Boissieu) H. Wolff	Zhou et al. (2008)	EU236213
*Trachydiumroylei* Lindl.	Valiejo-Roman et al. (2012)	FJ469972 & FJ483510

The Plant Genomic DNA Kit (Tiangen Biotech) was used to isolate DNA from materials of silica-gel-dried and herbarium specimens, and nuclear ribosomal DNA internal transcribed spacer (ITS) sequences were used for phylogenetic inference. Detailed information on PCR amplification and sequencing strategies was obtained from Zhou et al. (2008). Phylogenetic analyses were conducted using both Maximum Likelihood (ML) and Maximum Parsimony (MP). ML analyses were performed using MEGA7 (Kumar et al. 2016), with the GTR + G + I model and 1000 bootstrap (BS) replicates. Parsimony analysis was performed using PAUP* v. 4.0b10 (Swofford 2003). For the heuristic search, 100 random addition sequence replicates, tree bisection-reconnection (TBR), saving multiple trees and ACCTRAN optimisation were chosen. Gaps were treated as missing data. Bootstrap values were calculated from 1000 replicate analyses using TBR branch swapping and simple stepwise-addition of taxa.

## Results

### Molecular phylogenetic analysis

**Figure 2.**
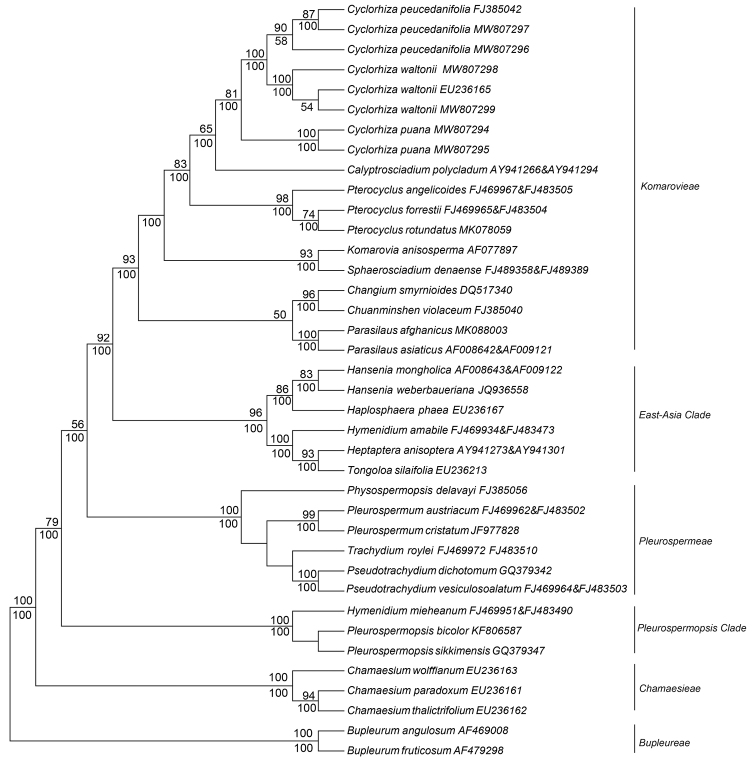
Phylogenetic tree derived from nrDNA ITS sequences, with support values in Maximum Likelihood and Maximum Parsimony above and below nodes, respectively.

The final aligned data matrix contained 629 positions, in which 256 were parsimony informative. The new species yielded high sequence divergence values with the other two species of the genus, i.e. *C.waltonii* (5.21%) and *C.peucedanifolia* (4.39–4.60%). The phylogeny showed that two accessions of the new species formed a strongly-supported monophyletic group and constituted a sister branch of *Cyclorhiza* species within the tribe Komarovieae (Fig. 2). Each of the three species was resolved as monophyletic groups, with the infraspecific divergence values ranging between 0.00–0.58%.

### Taxonomic treatment

#### Cyclorhiza puana

Taxon classificationPlantaeApialesApiaceae

J. Zhou & Z.W. Liu sp. nov.

E7033220-F982-5821-9D9C-6FDF6C6FC716

urn:lsid:ipni.org:names:77219738-1

Fig. 1G–I

##### Type.

China. Sichuan: Luhuo County, Renda Town, 3052 m, 100°38'59.57"E, 31°24'50.76"N, 17 Aug 2016, *J. Zhou, Z.W. Liu & Y.Z. Gao LZ201606120* (holotype: KUN! [KUN1519999]; isotype: KUN!).

##### Diagnosis.

*Cyclorhizapuana* resembles *C.waltonii* but differs from the latter in its long-cylindric roots with sparse annular scars (vs. stout, branched near stem into a cluster of several long, woody, carrot-like roots with prominent annular scars), smaller ultimate segments 2–4 × 0.5–1 mm (vs. 4–20 × 2–6 mm), rays subequal (vs. unequal), stylopodium obconic (vs. low-conic) and seed face slightly concave (vs. deeply sulcate).

##### Description.

Herbs perennial, 40–60 cm tall, glabrous. Taproots long-cylindrical with sparse annular scars. Stem base covered in purplish-brown remnant sheaths, solitary or rarely several, ribbed, unbranched or upper 1–3-branched, 2–3 mm in diameter. Basal and lower leaves petiolate, petioles 2–6 cm long, sheaths narrow, short; blade triangular-ovate in outline, 4-pinnatisect, 2–5 × 7–12 cm, ultimate segments linear, 2–4 × 0.5–1 mm. Upper leaves smaller and reduced. Umbels loose, compound, terminal and lateral; bracts absent or sometimes 1; bracteoles absent or rarely 1–2, linear; rays 4–6, subequal; umbellules 6–14-flowered, pedicels 6–8 mm, subequal. Calyx teeth minute, triangular; petals not known; stylopodium obconic, brown; styles short. Fruit oblong, 5 × 2 mm, dark yellow; ribs 5, filiform, prominent, slightly thickened; vittae 1 in each furrow, 2 on commissure. Seed face slightly concave. Carpophore 2-cleft to base.

##### Etymology.

The species epithet “puana” is given in honour of Prof. Pu Fading (1936–) for his outstanding contributions to the Chinese Apiaceae.

##### Vernacular name.

The Chinese name is given as “炉霍环根芹” (lú huò huán gēn qín), referring to the locality where the type specimen was collected.

##### Phenology.

Flowering from June to July, and fruiting from July to September.

##### Distribution and habitat.

**Figure 3.**
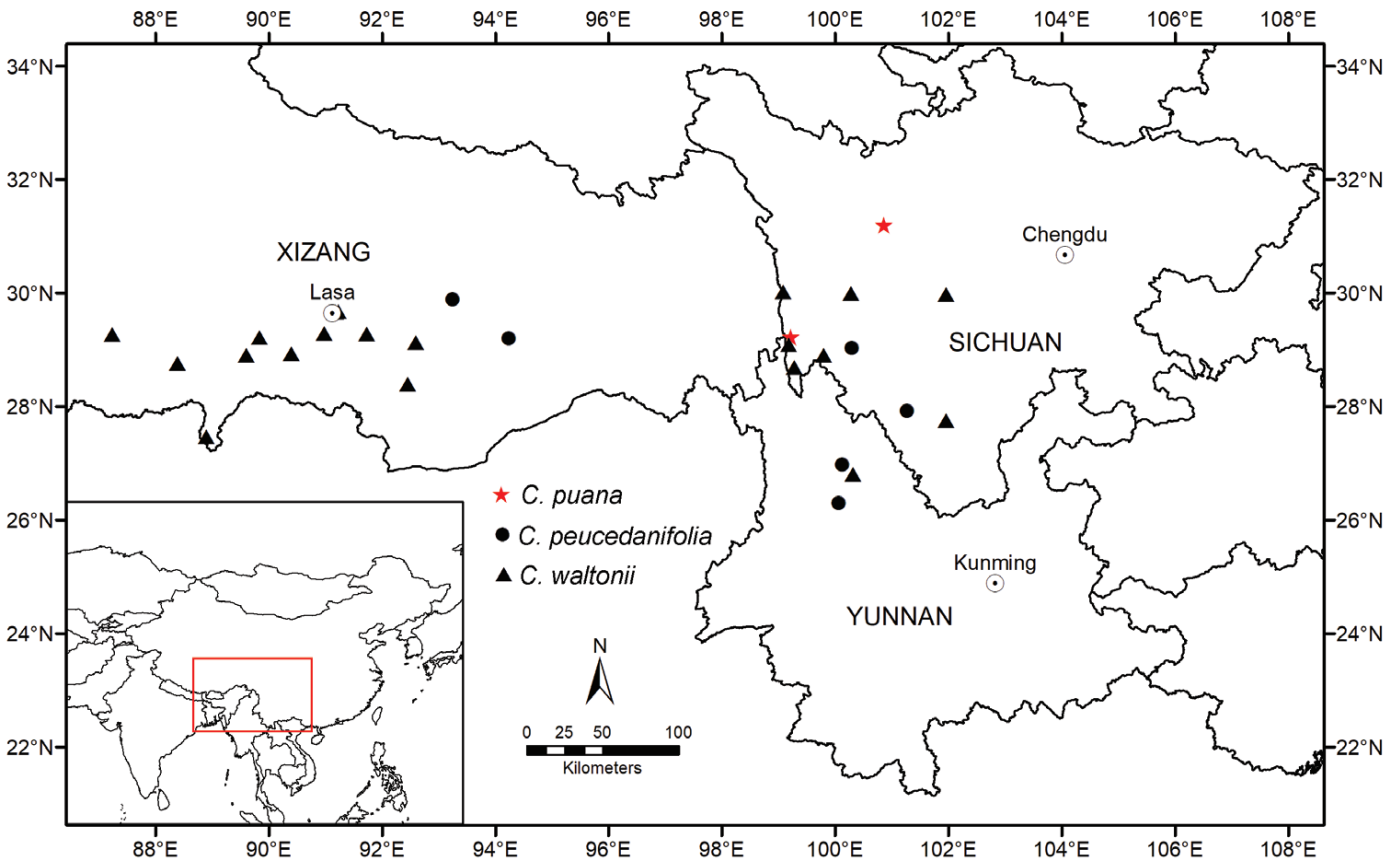
Distribution map of *C.waltonii*, *C.peucedanifolia* and *C.puana*.

The new species is distributed in Sichuan Province, China. It grows in the alpine open mixed forests at elevations of 3000–3200 m (Fig. 3).

##### Additional specimens examined (paratype).

China. Sichuan: Batang County, Jiangbading Village, 3268 m, 99°11'51"E, 29°55'54"N, 30 Jul. 2014, *X.X. Zhu, B. Chen, B. Shen &Y.G. Song CSH06561* (CSH! [CSH0037273]).

##### Conservation status.

So far, only two populations with no more than ten individuals have been found. Through further investigations, more populations may be discovered to assess its conservation status. Based on the available data, the new species can be assessed as Data Deficient (DD) on the basis of recommendations of the International Union for Conservation of Nature (IUCN 2019).

### Key to species of *Cyclorhiza*

**Table:** 

1	Ultimate leaf segments ovate-oblong to linear-lanceolate, 20–60 × 3–10 mm	*** C. peucedanifolia ***
–	Ultimate leaf segments linear, 2–20 × 0.5–6 mm	**2**
2	Ultimate leaf segments 4–20 × 2–6 mm; rays unequal; stylopodium low-conic; seed face deeply sulcate	*** C. waltonii ***
–	Ultimate leaf segments 2–4 × 0.5–1 mm; rays subequal; stylopodium obconic; seed face slightly concave	*** C. puana ***

## Discussion

Due to its topographical and climatic heterogeneity, the Hengduan Mountains of the Sino-Himalayas is one of the richest regions across China in terms of biodiversity (Ying and Zhang 1984; Pu 1993). These mountains harbor an incredible number of endemic species, among which three species of *Cyclorhiza* are found. *Cyclorhizawaltonii* is found in open broad-leaved forests, scrub and alpine meadows of W Sichuan, SE Xizang and NW Yunnan Provinces at elevations of 2500–4600 m. *Cyclorhizapeucedanifolia* occurs in alpine open mixed forests, bamboo thickets and scrub of SW Sichuan (Muli), SE Xizang, NW Yunnan Provinces at elevations of 1800–3600 m (Sheh and Watson 2005). The new species seems to be relatively rare and known only from two localities in Sichuan Province, where it grows in alpine open mixed forests at elevations of 3000–3200 m. Generally, the three species share a similar ecology and habitat.

*Cyclorhiza* has been regarded as a well-defined genus since it was established by Sheh and Shan in 1980. The new species possesses typical characteristics of the genus, such as taproots with prominent annular scars, bracts and bracteoles usually absent, and fruits subpentagonal in cross section, with variation in some characters (e.g. the seed face slightly concave, and the stylopodium obconic).

The specimen from CSH identified as *Cyclorhizawaltonii* actually corresponds to *C.puana*. The confusion between these two species is likely driven by the similar morphology of the leaf blades, i.e., ultimate segments. However, the ultimate leaf segments in *C.puana* are smaller, 2–4 × 0.5–1 mm, whereas those in *C.waltonii* are 4–20 × 2–6 mm (Sheh and Shan 1980). Additionally, *C.puana* can be distinguished by obconic stylopodium (vs. low-conic), ribs filiform, prominent, slightly thickened (vs. filiform, prominent, narrowly winged) and seed face slightly concave (vs. deeply sulcate). The genetic difference between these two species is relatively large (> 5%), further supporting their separate status.

## Supplementary Material

XML Treatment for Cyclorhiza puana
